# Antimicrobial properties of a novel copper-based composite coating with potential for use in healthcare facilities

**DOI:** 10.1186/s13756-018-0456-4

**Published:** 2019-01-05

**Authors:** David A. Montero, Carolina Arellano, Mirka Pardo, Rosa Vera, Ricardo Gálvez, Marcela Cifuentes, María A. Berasain, Marisol Gómez, Claudio Ramírez, Roberto M. Vidal

**Affiliations:** 1 Programa de Microbiología y Micología, Instituto de Ciencias Biomédicas, Facultad de Medicina, Universidad de Chile, Santiago, Chile; 2 Instituto de Química, Facultad de Ciencias, Pontificia Universidad Católica de Valparaíso, Valparaíso, Chile; 3 Unidad de Cuidados Intensivos, Facultad de Medicina, Hospital Clínico Universidad de Chile, Santiago, Chile; 4 ATACAMALAB, Lampa, Chile; 5 Instituto Milenio de Inmunología e Inmunoterapia, Facultad de Medicina, Universidad de Chile, Santiago, Chile

**Keywords:** Antimicrobial copper, Copper-based composite, Self-sanitizing coating, High-touch surfaces, Healthcare-associated infections

## Abstract

**Background:**

Healthcare-associated infections (HAIs) have a major impact on public health worldwide. Particularly, hospital surfaces contaminated with bacterial pathogens are often the origin of both sporadic cases and outbreaks of HAIs. It has been demonstrated that copper surfaces reduce the microbial burden of high touch surfaces in the hospital environment. Here we report the antimicrobial characterization of a novel composite coating with embedded copper particles, named Copper Armour™.

**Methods:**

The Copper Armour™ bactericidal activity was evaluated in in vitro assays against several bacterial pathogens, including *Staphylococcus aureus*, *Pseudomonas aeruginosa*, *Escherichia coli* O157:H7 and *Listeria monocytogenes.* Additionally, its antimicrobial properties were also evaluated in a pilot study over a nine-week period at an adult intensive care unit. For this, four high touch surfaces, including bed rails, overbed table, bedside table and IV Pole, were coated with Cooper Armour™, and its microbial burden was determined over a nine-week period.

**Results:**

Copper Armour™ coated samples showed an in vitro reduction in bacterial burden of > 99.9% compared to control samples. Moreover, pilot study results indicate that Copper Armour™ significantly reduces the level of microbial contamination on high-touch surfaces in the hospital environment, as compared with standard surfaces.

**Conclusions:**

Based on its antimicrobial properties, Copper Armour™ is a novel self-sanitizing coating that exhibits bactericidal activity against important human pathogens and significantly reduces the microbial burden of hospital surfaces. This composite could be used as a self-sanitizing coating to complement infection control strategies in healthcare facilities.

## Background

Healthcare-associated infections (HAIs) are the most frequent adverse event threatening the life of hospitalized patients worldwide [1]. HAIs have a major impact on public health, as they increase the average length of hospital stays, morbidity and mortality [2, 3], and cause a significant increase in healthcare costs [4, 5].

Multiple factors contribute to the incidence of HAIs, including intrinsic patient conditions (e.g. their individual pathologies) and risk factors associated with the hospital environment. Specifically, medical devices and hospital surfaces contaminated with pathogenic microorganisms are often the origin of both sporadic cases and outbreaks of HAIs [2, 6, 7]. Pathogens, such as methicillin-resistant *Staphylococcus aureus* (MRSA), vancomycin-resistant *Enterococcus* spp. (VRE) and *Clostridium difficile*, are able to colonize hospital surfaces, and both spores and the vegetative form can persist on these surfaces for months [7]. Therefore, hand hygiene and routine and terminal cleaning of surfaces in contact with the patients are useful strategies to limit intra-hospital propagation of infectious agents [8, 9]. At present, the microbiological standard used to evaluate and monitor terminal cleaning of hospital surfaces is a count of 250–500 aerobic colony-forming units (cfu) per 100 cm^2^ [10, 11]. However, while deep cleaning may remove the majority of microorganisms present on hospital surfaces, they are susceptible to recontamination, which in some cases occurs in a very short period of time [12].

In 2008, the United States Environmental Protection Agency (US EPA) recognized copper as the first antimicrobial metal. In in vitro assays, solid copper surfaces killed 99.9% of microorganisms within two hours of contact [13]. The rate of this antimicrobial activity has a magnitude of 7 to 8 logarithms per hour and generally no microorganisms are recovered after longer incubation periods [14]. Likewise, copper particles exhibit potent antimicrobial activity [15]. The bactericidal activity of copper is mainly attributed to the release of ions, which affect the integrity of the membrane and/or the bacterial wall, generate intracellular oxidative stress and are genotoxic, resulting in the death of microorganisms [14, 15]. One advantage of copper as a bactericidal agent is the low level of resistance among clinically relevant microorganisms. Copper-resistant mechanisms are primarily found in environmental microorganisms living in copper-rich niches, such as marine sediments and mines [15, 16].

Consequently, the number of studies evaluating the use of copper as a strategy for reducing the microbial burden in hospital environments and to prevent HAIs has increased in the past few years [11, 12, 17–23]. Results from these studies indicate that hospital surfaces coated with solid copper show sustained reduction in microbial burden compared to control surfaces. Nevertheless, additional studies are necessary to determine the impact of using copper-coated surfaces on the incidence of HAIs. While some studies concluded that using copper-coated surfaces reduces the rate of these infections [11, 22], in others this reduction was not statistically significant [21]. Furthermore, heterogeneity in study design and data analysis among existing studies makes it hard to compare their results, and therefore to draw definitive conclusions [24, 25]. Based on these observations, the use of copper-coated surfaces and medical devices is a promising strategy for controlling HAIs.

This report summarizes the development and antimicrobial characterization of a composite material that includes copper particles, named Copper Armour™. Due to its initial liquid state, this novel composite can be used to impregnate various surfaces; after it dries (~ 2.5 h) it provides a solid coating of 0.5–3.0 mm thick.

Many types of microorganisms can persist for extended periods of time on high-touch surfaces; therefore, this type of surfaces represent high risk spots for pathogen transmission and HAIs. In this context, a main concern is to eliminate as many pathogenic microorganisms as possible from these surfaces and limiting their transfer to patients [26] . Due to their antimicrobial properties, metals, including copper, have been a focus of interest as coating materials for surfaces. In this scenario, the aim of this study was to determine in situ whether a composite based on copper significantly reduced the microbial load on coated surfaces in an adult intensive care unit (ICU) when compared to control (i.e. non-coated) surfaces and, in parallel, to determine if a composite based on copper has in vitro antimicrobial activity against relevant pathogenic bacteria.

## Methods

### Bacterial strains and culture conditions

The microorganisms used in this study were obtained from American Type Culture Collection (ATCC) and they include: *Staphylococcus aureus* (ATCC 29213), *Pseudomonas aeruginosa* (ATCC 27853), *Escherichia coli* O157:H7 (ATCC 43895) and *Listeria monocytogenes* (ATCC 13932). *S. aureus*, *P. aeruginosa* and *E. coli* were routinely cultured in Trypticase Soy Broth (TSB, BD Difco™, USA) and Trypticase Soy Agar (TSA, BD Difco™, USA) for 24–36 h at 37 ± 0.5 °C. *L. monocytogenes* was routinely cultured in Brain Heart Infusion Broth (BHI, BD Difco™, USA) and BHI Agar (BD Difco™, USA) for 24–48 h at 37 ± 0.5 °C.

### Formulation of copper Armour™

**Fig. 1:**
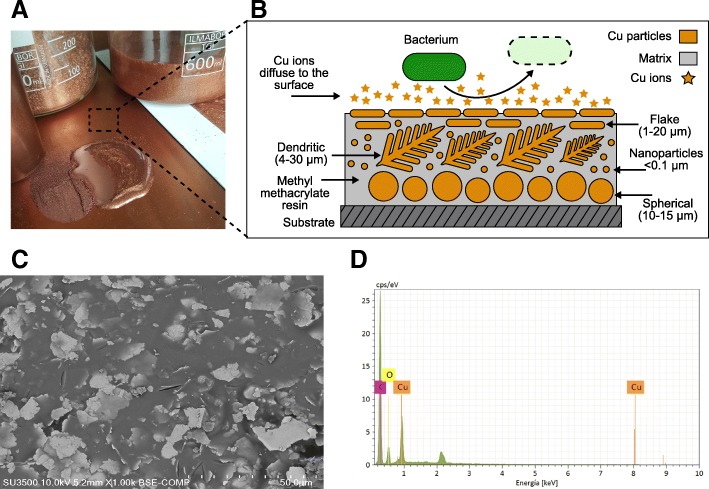
Formulation of Copper Armour™. **a** Copper Armour™ can be applied in liquid state on various substrates. At 25 °C, a 1 mm thick coating requires 2.5 h to dry. **b** Schematic composition of Copper Armour™. Shapes and sizes of Cu particles embedded in the methacrylate resin (matrix) are shown; the matrix acts as a liquid medium, providing adherence to the substrate and cohesion among components. Larger spherical Cu particles precipitate before curing of the matrix. Dendritic Cu particles act as a charge-conducting network. Smaller flakes Cu particles, float on the surface and become oriented in parallel, increasing the contact surface, thus, favoring the release of Cu ions. A bacterium is shown with its membrane degraded as a consequence of the antimicrobial activity of Cu. **c** Superficial topography of Copper Armour™. SEM analysis showed a homogenous distribution of copper particles in the matrix. **d** Chemical composition of Copper Armour™. EDAX analysis shows that Cu, carbon (C) and oxygen (O) are the main elements of the composite

Copper Armour™ is a composite material that is embedded with copper particles in a methyl methacrylate resin (matrix) evenly distributed in the matrix, so that copper particles are always partially exposed on the surface. To achieve this effect, at least four types of copper particles are used; as these particles differ in shape, apparent densities (with a range of < 1–8 g/cm^3^; Fig. 1a, b) and capacity to be compacted among themselves, when mixed together in a polymeric matrix they can be distributed homogeneously in the entire thickness of the composite structure.

Three components were separately formulated. The first component, a polymeric base, includes an agglomerative or polymeric matrix with a dispersion of copper nanoparticles and microparticles < 20 μm; this dispersion is achieved by conventional methods using a high-shear mixing blade (Cowles), where nanoparticles < 0.1 μm were previously homogenized by an ultrasonic agitator. This semi-manufactured product is filtered using a 200-mesh (74 μm) sieve prior to packaging. The second component, the active component, is made from larger copper particles, up to 60 μm, that are dry packaged after sieving and drying to avoid agglomeration. The third component, a hardener, is separately packaged in a third container, depending on the selected agglomerate.

The application of Copper Armour™ is performed using a disperser, preferably electric from 200 to 600 rpm, homogenizing the polymeric base with the active component. Then, the hardener is added and homogenized for at least one minute. This mixture must be applied within 10 min following preparation, as after 15 min it will begin to solidify.

For the assays described in this work, the Copper Armour™ formulation correspond to a 60/40 copper/agglomerate total weight ratio. The agglomerative methyl methacrylate resin used was DEGADUR 527 (Evonik A.G., Germany), with powdered solid peroxide hardener. Copper Armour™ formulations are protected by Patent Cooperation Treaty international application number: PCT/CL2015/050058.

### Electron microscopy

The superficial topography of Copper Armour™ was analyzed by scanning electron microscopy (SEM) using a Hitachi SU 3500 microscope coupled to a series 410-M detector, which allowed us to qualitatively analyze the elements present by Energy Dispersive X-ray spectroscopy (EDAX). Samples were coated with gold (Au) to render them conductive.

### In vitro evaluation of antimicrobial activity

The in vitro evaluation of antimicrobial activity was conducted based on two EPA protocols [27, 28], with slight modifications. The EPA designed these protocols to determine the efficacy of copper as a disinfectant, and to quantify the continuous reduction of bacterial contamination of non-porous surfaces containing copper and its alloys.

#### Test method of sanitizer activity (protocol 1)

Two batches of test samples (each one consisting of five 2 × 2 cm aluminum sheets coated with Copper Armour™) and ten control samples (2 × 2 cm aluminum sheets) were evaluated per microorganism. Test and control samples were cleaned using 70% ethanol and washed using sterile distilled water. Each sample was placed in a Petri dish and allowed to dry in a biological safety cabinet (Class II type A2, NuAire, USA), followed by exposure to ultraviolet light for 15 min per side.

Bacterial culture media were supplemented with 5% heat-inactivated fetal calf serum (GIBCO, USA) and 0.01% Triton X-100 as organic sediment load. Initial inocula (10^7^ to 10^8^ cfu) were determined by serial dilutions in 1X phosphate-buffered saline (PBS) and plated in duplicate on TSA for 24–48 h at 37 ± 0.5 °C. Test samples and controls were inoculated with 0.02 ml of bacterial culture spread over ~ 0.3 cm^2^ and allowed to dry for 20–40 min. After 60 min of exposure (at room temperature) to the challenging microorganisms, samples were transferred to 20 ml of neutralizing solution [TPL; Trypticase Soy Broth plus Polysorbate 80 (1.5% *v*/v) and Lecithin (0.07% v/v)], sonicated in an ultrasonic bath (Neytech ultrasonic cleaner, Model 19H, USA) for 5 min and turned to mix. Within 1 h, serial dilutions were performed in PBS and plated in duplicate on TSA. After incubation for 24–48 h at 37 ± 0.5 °C, the number of cfu was counted. The number of cfu recovered per sample was determined taking into consideration the dilution (20x), using the following equation: cfu/sample = (A x D x V) / V_2_, where A = average cfu per sample, counted in duplicate; D = dilution factor; V = volume of TPL solution added; and V_2_ = volume plated. The percentage reduction in the number of cfu for test samples as compared with the control samples was determined using the following equation: % reduction = [(a-b) / a] × 100 where, a = geometric median of the number of cfu recovered in control samples; and b = geometric median of the number of cfu recovered in the test samples.

In addition, the following sterility control was performed: 0.1 ml aliquots of culture media, PBS and TPL solution were plated on TSA and the absence of bacterial growth was confirmed. One test and one control sample, sterilized as previously described, were washed using 1 ml of TPL solution, 0.1 ml of this solution was plated on TSA and the absence of bacterial growth was confirmed. Finally, each microorganism was inoculated in 1 ml of TPL solution, and it was determined that this solution did not inhibit bacterial growth.

#### Test method of continuous reduction of bacterial contamination (protocol 2)

Two batches of test samples (each one consisting of three 2 × 2 cm aluminum sheets coated with Copper Armour™) and six control samples (2 × 2 cm aluminum sheets) were evaluated per microorganism in a similar fashion to that described in Protocol 1. Samples were consecutively inoculated eight times, adding the challenging microorganism at 0, 3, 6, 9, 12, 15, 18 and 21 h. The antimicrobial efficacy was evaluated at 2, 6, 12, 18 and 24 h, corresponding to 1, 2, 4, 6 and 8 inoculations. After exposure to bacteria, 20 ml of TPL solution was added and samples were subjected to sonication in an ultrasonic bath and turned to mix. The determination of the number of cfu recovered per sample and the percent reduction was performed as described for protocol 1. Additionally, we performed the same sterility controls as previously described.

### Pilot study at an adult intensive care unit

**Fig. 2:**
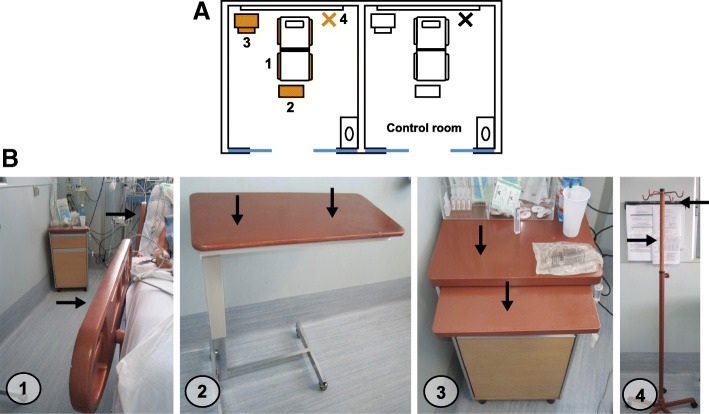
Distribution of coated and sampled surfaces within the adult intensive care unit rooms. **a** Distribution of the sampled objects within the room. In the intervention room, the coated surfaces are shown in gold. **b** Copper Armour™ coated objects**.** (1) Bed rails, (2) Overbed table, (3) Bedside table and (4) IV Pole. Black arrows indicate where surface sampled were taken for each object

The study was conducted in two patient rooms (side by side) within the adult ICU at the Hospital Clínico Universidad de Chile located in Santiago, Chile. One of the rooms was defined as the control and in the other room, considered the intervention room, surfaces were coated with Copper Armour™ (Fig. 2a). The following surfaces were coated with Copper Armour™: bed rails, overbed table, bedside table and IV Pole (Fig. 2b). Upon admission, patients were randomly assigned to either the control or intervention (Copper Armour™) room. Hand hygiene and cleaning protocols remained unaltered during the study.

The sampling protocol was performed over a nine-week period, during which the first week (basal week) was dedicated to methodology adjustments. Data obtained during this week were not included in statistical analysis and are not shown. Rooms were sampled on the same day and at the same time (before morning cleaning) every week throughout the study. Surfaces were sampled in duplicate (Fig. 2b, black arrows) using sterile plastic templates of 2 × 12.5 cm, in the case of bed rails the IV Pole, or 5 × 5 cm, in the case of the overbed and bedside tables. PBS humidified sterile dressing was vigorously scrubbed 10 times horizontally and 10 times vertically, covering the whole sampling area (25 cm^2^). Each dressing was placed in a 50 ml sterile polypropylene centrifuge tube. Within 2 h, three ml of PBS/LT (0.5% Tween 80 and 0.07% lecithin) were added to each centrifuge tube, vortexed for 1 min, and allowed to settle for 5 min. Subsequently, 100 μl aliquots were plated on 5% sheep blood agar to estimate the total aerobic microbial burden present on sampled surface; mannitol salt agar (BD Difco™, USA) to estimate the number of cfu of *Staphylococcus* spp.; MacConkey agar (BD Difco™, USA) to determine the number of cfu of Gram-negative bacilli; chromogenic agar (BBLTM-BD CHROMagar MRSA™, Becton Dickinson, USA) to estimate the number of cfu of MRSA; bile esculin agar (Becton Dickinson, USA) supplemented with vancomycin (6 μg/ml) to determine the number of cfu of VRE and Sabouraud agar (Becton Dickinson, USA) supplemented with chloramphenicol (CAF) to estimate the cfu of yeast / fungi. Plates were incubated for 24–48 h at 37 ± 0.5 °C and the number of cfu were determined. The number of cfu recovered per sample was reported as cfu/100 cm^2^.

### Statistical analysis

Data on microbial burden obtained from hospital surfaces were analyzed for normality using the Shapiro-Wilk test. As the data did not follow a normal distribution, the non-parametric Mann-Whitney U test (one-tailed) was used to determine if the microbial burden of Copper Armour™ coated surfaces was significantly lower compared to control surfaces. Additionally, differences in the frequency of microbial burden, reported as > 250 cfu/100 cm^2^ surface, between control and Copper Armour™ coated surfaces was analyzed using the Fisher’s exact test or the Pearson χ^2^ test (if all expected frequencies were ≥ 5). A *P*-value of < 0.05 was considered statistically significant; statistical analysis was performed in GraphPad Prism version 6.00 (GraphPad Software, La Jolla California USA).

## Results

### Characterization of the microstructure and chemical composition of copper Armour™

SEM analysis of samples coated with Copper Armour™ showed a homogenous distribution of copper particles in the methacrylate matrix (Fig. 1c). Additionally, qualitative chemical analysis indicated that the main component in the coating was copper (Cu), while carbon (C) and oxygen (O) were the main matrix components (Fig. 1d).

### In vitro evaluation of the antimicrobial properties of copper Armour™

Challenging microorganisms for the evaluation of in vitro bactericidal activity were *Staphylococcus aureus*, *Pseudomonas aeruginosa*, *Escherichia coli* O157:H7 and *Listeria monocytogenes*. All experiments conducted with Copper Armour™ coated samples showed a reduction, after 1 h of contact, in bacterial burden of > 99.9% compared to control samples (Table 1). Additionally, we determined that after consecutive inoculations over 24 h, Copper Armour™ coated samples continued to reduce the microbial burden by > 99.9% compared to control samples (Table 2). Thus, Copper Armour™ continuously reduced contamination caused by the bacteria evaluated here.

**Table 1:** Reduction in bacterial burden after 1 h of contact with Copper Armour™ as compared to control surfaces

Microorganism	Batch	Inoculum (cfu)	Number of cfu recovered per sample *		Reduction (%) **
			Control	Copper ArmourTm	
*S. aureus*	1	4.3 × 10^7^	1.2 × 10^6^; 3.0 × 10^6^; 1.8 × 10^6^; 1.9 × 10^6^; 3.1 × 10^6^	< 1;< 1;< 1;< 1;< 1	> 99.9
	2	1.5 × 10^7^	1.4 × 10^6^; 1.1 × 10^6^; 1.1 × 10^6^; 2.0 × 10^6^; 1.2 × 10^6^	< 1;< 1;< 1;< 1;< 1	> 99.9
*P. aeruginosa*	1	1.6 × 10^8^	4.4 × 10^7^; 2.1 × 10^7^; 7.2 × 10^6^; 4.4 × 10^7^; 9.3 × 10^6^	< 1;< 1;< 1;< 1;< 1	> 99.9
	2	1.8 × 10^8^	1.1 × 10^7^; 2.8 × 10^7^; 1.2 × 10^7^; 1.0 × 10^7^; 1.1 × 10^7^	< 1;< 1;< 1;< 1;< 1	> 99.9
*E. coli* O157:H7	1	1.9 × 10^7^	8.1 × 10^5^; 4.3 × 10^6^; 4.1 × 10^6^; 5.4 × 10^6^; 9.6 × 10^5^	< 1;< 1;< 1;< 1;< 1	> 99.9
	2	2.4 × 10^7^	5.3 × 10^6^; 3.8 × 10^6^; 2.4 × 10^6^; 2.5 × 10^6^; 7.9 × 10^5^	< 1;< 1;< 1;< 1;< 1	> 99.9
*L. monocytogenes*	1	3.2 × 10^7^	7.2 × 10^6^; 8.7 × 10^6^; 9.4 × 10^6^; 7.3 × 10^6^; 6.3 × 10^6^	< 1;< 1;< 1;< 1;< 1	> 99.9
	2	1.6 × 10^7^	9.7 × 10^6^; 8.0 × 10^6^; 7.3 × 10^6^; 7.7 × 10^6^; 7.8 × 10^6^	< 1;< 1;< 1;< 1;< 1	> 99.9

* Each value corresponds to the average of duplicates of cfu recovered in each one of the five samples evaluated per production batch. ** As compared with control samples

**Table 2:** Continuous reduction of bacterial burden over 24 h of contact with Copper Armour™ as compared to control surfaces

Microorganism	Time (h)	Batch	Number of cfu recovered per sample *		Reduction (%) **
			Controls	Copper ArmourTm	
*S. aureus* Inoculum: 2.0 × 10^7^–5.0 × 10^7^	2	1 2	3.8 × 10^5^; 3.1 × 10^5^; 3.9 × 10^5^ 4.0 × 10^5^; 4.1 × 10^5^; 3.2 × 10^5^	< 1;< 1;< 1 < 1;< 1;< 1	> 99.9
	6	1 2	1.8 × 10^6^; 1.8 × 10^6^; 2.0 × 10^6^ 1.1 × 10^6^; 1.5 × 10^6^; 1.2 × 10^6^	< 1;< 1;< 1 < 1;< 1;< 1	> 99.9
	12	1 2	4.4 × 10^6^; 4.5 × 10^6^; 4.5 × 10^6^ 3.9 × 10^6^; 4.4 × 10^6^; 4.0 × 10^6^	< 1;< 1;< 1 < 1;< 1;< 1	> 99.9
	18	1 2	6.6 × 10^6^; 5.9 × 10^6^; 6.1 × 10^6^ 7.9 × 10^6^; 6.4 × 10^6^; 6.8 × 10^6^	< 1;< 1;< 1 < 1;< 1;< 1	> 99.9
	24	1 2	2.0 × 10^7^; 1.0 × 10^7^; 1.3 × 10^7^ 1.0 × 10^7^; 9.4 × 10^6^; 9.9 × 10^6^	< 1;< 1;< 1 < 1;< 1;< 1	> 99.9
*P. aeruginosa* Inoculum: 1.6 × 10^8^–1.8 × 10^8^	2	1 2	7.4 × 10^6^; 7.4 × 10^6^; 7.2 × 10^6^ 6.2 × 10^6^; 6.8 × 10^6^; 6.5 × 10^6^	< 1;< 1;< 1 < 1;< 1;< 1	> 99.9
	6	1 2	7.6 × 10^6^; 7.8 × 10^6^; 7.6 × 10^6^ 8.2 × 10^6^; 7.8 × 10^6^; 7.9 × 10^6^	5800; < 1; 2000 < 1;< 1;< 1	> 99.9
	12	1 2	1.6 × 10^7^; 1.4 × 10^7^; 1.3 × 10^7^ 1.0 × 10^7^; 1.1 × 10^7^; 2.0 × 10^7^	< 1;< 1;< 1 < 1;< 1;< 1	> 99.9
	18	1 2	4.8 × 10^7^; 4.8 × 10^7^; 4.3 × 10^7^ 5.0 × 10^7^; 5.2 × 10^7^; 4.9 × 10^7^	< 1;< 1;< 1 < 1;< 1;< 1	> 99.9
	24	1 2	1.1 × 10^8^; 1.0 × 10^8^; 9.6 × 10^7^ 1.3 × 10^8^; 2.0 × 10^8^; 1.9 × 10^8^	< 1;< 1;< 1 < 1;< 1;< 1	> 99.9
*E. coli* O157:H7 Inoculum: 2.0 × 10^7^–4.0 × 10^7^	2	1 2	2.8 × 10^5^; 3.1 × 10^5^; 3.0 × 10^5^ 3.5 × 10^5^; 3.5 × 10^5^; 3.3 × 10^5^	< 1;< 1;< 1 < 1;< 1;< 1	> 99.9
	6	1 2	1.6 × 10^6^; 1.7 × 10^6^; 1.6 × 10^6^ 2.2 × 10^6^; 1.9 × 10^6^; 2.0 × 10^6^	< 1;< 1;< 1 < 1;< 1;< 1	> 99.9
	12	1 2	4.6 × 10^6^; 4.6 × 10^6^; 4.5 × 10^6^ 4.2 × 10^6^; 4.9 × 10^6^; 4.3 × 10^6^	< 1;< 1;< 1 < 1;< 1;< 1	> 99.9
	18	1 2	9.8 × 10^6^; 1.1 × 10^7^; 9.5 × 10^6^ 1.2 × 10^7^; 1.0 × 10^7^; 1.0 × 10^7^	< 1;< 1;< 1 < 1;< 1;< 1	> 99.9
	24	1 2	3.2 × 10^7^; 3.0 × 10^7^; 2.9 × 10^7^ 3.9 × 10^7^; 4.1 × 10^7^; 4.0 × 10^7^	< 1;< 1;< 1 < 1;< 1;< 1	> 99.9
*L. monocytogenes* Inoculum: 1.0 × 10^7^–5.0 × 10^7^	2	1 2	1,6 × 10^7^; 2,1 × 10^7^; 2,2 × 10^7^ 1,5 × 10^7^; 1,4 × 10^7^; 2,1 × 10^7^	< 1;< 1;< 1 < 1;< 1;< 1	> 99.9
	6	1 2	3,0 × 10^7^; 4,1 × 10^7^; 4,3 × 10^7^ 3,6 × 10^7^; 3,7 × 10^7^; 2,7 × 10^7^	< 1;< 1;< 1 < 1;< 1;< 1	> 99.9
	12	1 2	4,8 × 10^7^; 5,1 × 10^7^; 6,0 × 10^7^ 4,7 × 10^7^; 4,7 × 10^7^; 4,1 × 10^7^	< 1;< 1;< 1 < 1;< 1;< 1	> 99.9
	18	1 2	9,8 × 10^7^; 9,1 × 10^7^; 9,8 × 10^7^ 9,2 × 10^7^; 9,4 × 10^7^; 9,0 × 10^7^	< 1;< 1;< 1 < 1;< 1;< 1	> 99.9
	24	1 2	2,2 × 10^8^; 2,0 × 10^8^; 2,1 × 10^8^ 1,9 × 10^8^; 1,2 × 10^8^; 1,2 × 10^8^	< 1;< 1;< 1 < 1;< 1;< 1	> 99.9

* Each value corresponds to the average of duplicates of cfu recovered in each one of the three samples evaluated per production batch. ** As compared with control samples

### Evaluation of the antimicrobial properties of copper Armour™ at an adult intensive care unit

Copper Armour™ coated surfaces (Fig. 2) showed a reduction of the aerobic microbial burden compared to control surfaces; this reduction was statistically significant for bed rails (66%; *p* = 0.018) and the overbed Table (56%; *p* = 0.045). Additionally, the average number of cfu/100 cm^2^ for *Staphylococcus* spp. was lower on Copper Armour™ coated surfaces compared to control surfaces (Table3); however, this reduction was only statistically significant in the case of bed rails (88.9%; *p* < 0.001). It is important to mention that during the study *S. aureus* was not recovered from any surface, and only one Copper Armour™ coated surface was positive for Gram negative bacilli (720 cfu/100 cm^2^) and 2 for VRE (both samples with 120 cfu/100 cm^2^); due to these low detection rates, these microorganisms were not included in statistical analyses. In contrast, we did not observe a reduction in the average burden of yeasts / fungi on Copper Armour™ coated surfaces as compared to control surfaces. Nevertheless, the isolation of these microorganisms was sporadic overall, with values < 250 cfu/100 cm^2^ during the study.

**Fig. 3:**
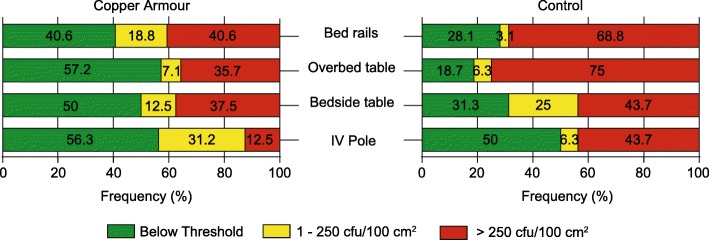
Frequency distribution of microbial burden on Copper Armour™ coated surfaces and control surfaces. The microbial burden observed for each sample was classified into three categories: below the detection threshold (green), 1 to 250 cfu/100 cm^2^ (yellow) or > 250 cfu/100 cm^2^ (red)

In agreement with previous results, the frequency of samples with a microbial burden > 250 cfu/100 cm^2^ was lower in the case of Copper Armour™ coated surfaces compared to control surfaces (Fig. 3); this difference was statistically significant for bed rails (40.6% Copper Armour™ versus 68.8% control; *p* = 0.023) and the overbed Table (35.7% Copper Armour™ versus 75% control; *p* = 0.030) (Table 4). Furthermore, the overall frequency of control surfaces with a microbial burden of > 250 cfu/100 cm^2^ was significantly greater than Copper Armour coated surfaces, 60% (48/80) versus 33.3% (*p* = 0.007). Thus, Copper Armour™ exhibits antimicrobial properties able to decrease the microbial burden of high-touch surfaces in a hospital environment. Therefore, compared to control surfaces, Copper Armour™ coated surfaces were more likely to meet the threshold required for successful terminal cleaning (i.e. < 250 cfu/100 cm^2^), indicating that the use of this composite could contribute to schemes and practices aimed at controlling HAIs.

## Discussion

It has been demonstrated that high-touch surfaces in the hospital environment are an important reservoir for infectious agents causing HAIs [6, 29]. In this context, a considerable number of studies have provided experimental evidence indicating that hospital surfaces coated with copper have lower microbial burden levels compared to standard surfaces, which in some cases have been associated with a reduction in the incidence of HAIs [11, 12, 17–23]. However, while most of these studies have been conducted using solid copper and its alloys, the in vitro and in situ evaluation of polymeric matrices and composites containing copper particles has been limited [30–33].

Our results indicate that Copper Armour™ may be used as a self-sanitizing coating to modify existing hospital surfaces, avoiding the structural restrictions imposed by a change to solid copper. Due to its initial liquid state and subsequent hardening, this composite can be used to coat surfaces of various sizes, shapes and compositions, which reduces the cost and quantity of copper required.

The in vitro evaluation of the antimicrobial properties of Copper Armour™ showed that this composite material exhibits a potent bactericidal activity against *S. aureus*, *P. aeruginosa*, *E. coli* O157:H7 and *L. monocytogenes*. As reported for solid copper, Copper Armour™ killed more than 99.9% of these microorganisms after one hour of contact, as well as after consecutive inoculations over 24 h (Table 1 & Table 2). It is noteworthy that two of these microorganisms, *S*. *aureus* and *P. aeruginosa,* are among the principal pathogens causing HAIs worldwide [34–36]. Moreover, the emergence of resistant and multiresistant bacteria makes it necessary to develop new biocidal materials and agents able to limit the dissemination and, at the same time, contribute to the elimination of these pathogens.

We also evaluated the Copper Armour™ antimicrobial properties in a hospital environment. Our pilot study indicated that Copper Armour™ reduces the microbial burden of hospital surfaces, even under present day protocols of extreme hygiene. A study by Attaway et al. [6] showed that bed rails in ICUs are rapidly colonized after cleaning with two commercial disinfectants, exceeding the threshold of 250 cfu/100 cm^2^ after 2.5 h. In that study, the average microbial burden found on bed rails before cleaning was 4.756 cfu/100 cm^2^ (median 1.665 cfu/100 cm^2^). Likewise, our results showed that control bed rails had an average microbial burden of 3.323 cfu/100 cm^2^ (median 1.440 cfu/100 cm^2^) (Table 3). On the contrary, Copper Armour™ coated bed rails showed an average microbial burden of 1.129 cfu/100 cm^2^ (median 120 cfu/100 cm^2^), which corresponds to a significant reduction (66%; *p* = 0.018) compared to control bed rails. Thus, Copper Armour™ exhibits antimicrobial properties able to decrease the microbial burden of high-touch surfaces in a hospital environment. Therefore, compared to control surfaces, Copper Armour™ coated surfaces were more likely to meet the threshold required for successful terminal cleaning (i.e. < 250 cfu/100 cm^2^), indicating that the use of this composite could contribute to schemes and practices aimed at controlling HAIs.

**Table 3:** Bacterial burden on Copper Armour™ coated surfaces and control surfaces during 8 weeks of pilot study in an adult intensive care unit

Evaluated object	Copper Armour™			Control			*P* value	% Reduction
	n	Average cfu/100 cm^2^	Media cfu/100 cm^2^	n	Average cfu/100 cm^2^	Median cfu/100 cm^2^		
Total aerobic microbial load								
Bed rails	32	1129	120	32	3323	1440	0.018 *	66.0
Overbed Table	14	762,9	0	16	1755	960	0,045 *	56.5
Bedside Table	16	1793	60	16	2108	120	0,303	14.9
IV Pole	16	157,5	0	16	337,5	120	0,195	53.5
*Staphylococcus* spp.								
Bed rails	32	270	0	32	2445	300	0,001 **	88.9
Overbed Table	14	462,9	0	16	720	240	0,106	35.7
Bedside Table	16	270	0	16	997,5	0	0,289	72.9
IV Pole	16	22,5	0	16	60	0	0,231	62.5
Yeasts/Fungi								
Bed rails	32	697,5	0	32	195,0	0	–	–
Overbed Table	14	68,5	0	16	15,00	0	–	–
Bedside Table	16	630	0	16	1155	0	0,279	45.5
IV Pole	16	15	0	16	37,5	0	0,367	60

* p < 0.05, ** p < 0.001 established using Mann-Whitney U test (one-tailed)

It must be noted that two previous studies demonstrated that bed rails of solid copper showed a significantly lower average microbial burden compared to control bed rails [12, 37]. Also, in agreement with our results, in those studies it was determined that *Staphylococcus* spp. were the main bacterial group contaminating ICU bed rails. In fact, Copper Armour™ coated bed rails showed a significant (88.9%, *p* < 0.001) reduction in the average burden of *Staphylococcus* spp. compared to control bed rails (Table 3).

The overbed table is another Copper Armour™ coated surface in which a significant reduction (56%, *p* = 0.045) of microbial burden was observed compared to the control overbed table. Besides, a lower average burden of *Staphylococcus* spp. was observed in the Copper Armour™ coated overbed table compared to the control overbed table, but in this case, the reduction, while showing a trend, was not significant (*p* = 0.105); this is likely due to the fact that the average burden of these microorganism on the control surface was also low. Previous studies have also shown that solid copper coated overbed tables have lower level of microbial burden compared to standard surfaces [37].

An intriguing result was the average microbial burden of the Copper Armour™ coated bedside tables compared to the control. In this case, only a small and non-significant reduction (*p* = 0.289) of contamination levels was observed (Table 3 & Table 4). A possible explanation for this result is that objects brought into the hospital, which escape cleaning schemes, are constantly placed on the bedside table (Fig. 2b).

**Table 4:** Frequency of a microbial burden of > 250 cfu/100 cm^2^ on Copper Armour™ coated surfaces and control surfaces

Evaluated objects	Copper Armour™		Control		P value
	n	Number (%) of samples having > 250 cfu/100 cm^2^	n	Number (%) of samples having > 250 cfu/100 cm^2^	
Bed rails	32	13 (40.6)	32	22 (68.8)	0.023 *
Overbed Table	14 **	5 (35.7)	16	12 (75)	0.030 *
Bedside Table	16	6 (37.5)	16	7 (43.7)	0.718
IV Pole	16	2 (12.5)	16	7 (43.7)	0.113
Total	78	26 (33.3)	80	48 (60)	0.001 **

* *p* < 0.05, ** *p* < 0.001 established using either Pearson χ^2^ or Fisher’s Exact tests

**Two samples were discarded because the surface was contaminated with blood

It has been reported that among the objects located within a patient’s room, the IV pole shows, in general, the lowest average microbial burden [18, 37]. This was also observed in the present study. It is likely that for this reason we were not able to observe differences between the average microbial burden of a Copper Armour™ coated IV pole and the control surface. Nevertheless, 87.5% (14/16) of the samples from the Copper Armour™ coated IV Pole showed levels < 250 cfu/100 cm^2^ as compared to a 56.3% (9/16) of control samples (Fig. 3). This suggests that, in the case of surfaces exposed to low levels of contamination, the main benefit provided by Copper Armour™ would be to extended protection time of the terminal cleaning.

The pilot study also attempted to investigate the antifungal properties of Copper Armour™. Nevertheless, we were not able to complete this aim as isolation of fungi/yeast was sporadic and with low numbers of cfu/100 cm^2^. Therefore, in order to evaluate this property, it would be necessary to implement a different methodological design.

Finally, our pilot study did not include parameters, such as whether the room was occupied / unoccupied each day or epidemiological data of the patients. Future studies, that are longer in duration and that include different hospital surfaces beyond those tested here, and that also consider patient factors are necessary to further evaluate the possible impact of Copper Armour™ on the incidence of HAIs.

## Conclusions

Our study suggests that Copper Amour TM, a novel self-sanitizing coating, exhibits bactericidal activity against important human pathogens and significantly reduces the microbial burden of hospital surfaces. Consequently, this novel composite could be used to complement infection control strategies in healthcare facilities.

## Abbreviations

ATCC: American Type Culture Collection
Au: Gold
BD: Becton Dickinson
cfu: colony-forming units
EDAX: Energy Dispersive X-ray spectroscopy
GIBCO: Grand Island Biological Company
HAI: Healthcare-associated infection
ICU: Intensive Care Unit
MRSA: Methicillin-Resistant Staphylococcus aureus
PBS: Phosphate-Buffered Saline
PBS/LT: Phosphate-Buffered Saline/Tween and Lecithin
PCT: Patent Cooperation Treaty
SEM: Scanning Electron Microscopy
TM: Trade Mark
TPL: Trypticase Soy Broth plus Polysorbate and Lecithin
TSA: Trypticase Soy Agar
TSB: Trypticase Soy Broth
US EPA: United States Environmental Protection Agency
VRE: Vancomycin-Resistant Enterococcus spp
